# PGC1α induced by reactive oxygen species contributes to chemoresistance of ovarian cancer cells

**DOI:** 10.18632/oncotarget.19140

**Published:** 2017-07-10

**Authors:** Boyun Kim, Je Won Jung, Jaeyoung Jung, Youngjin Han, Dong Hoon Suh, Hee Seung Kim, Danny N. Dhanasekaran, Yong Sang Song

**Affiliations:** ^1^ Cancer Research Institute, College of Medicine, Seoul National University, Seoul 03080, Korea; ^2^ Nano System Institute, Seoul National University, Seoul 08826, Korea; ^3^ Research Institute of Agriculture and Life Sciences, Seoul National University, Seoul 08826, Korea; ^4^ WCU Biomodulation, Department of Agricultural Biotechnology, Seoul National University, Seoul 08826, Korea; ^5^ Department of Obstetrics and Gynecology, Seoul National University Bundang Hospital, Seongnam 13620, Korea; ^6^ Stephenson Cancer Center, University of Oklahoma Health Sciences Center, Oklahoma City, OK 73012, United States of America; ^7^ Department of Obstetrics and Gynecology, College of Medicine, Seoul National University, Seoul 03080, Korea

**Keywords:** ovarian cancer, ROS, PGC1α, chemoresistance, mitochondria

## Abstract

Malignant cells are subjected to high levels of oxidative stress that arise from the increased production of reactive oxygen species (ROS) due to their altered metabolism. They activate antioxidant mechanisms to relieve the oxidative stress, and thereby acquire resistance to chemotherapeutic agents. In the present study, we found that PGC1α, a key molecule that both increases mitochondrial biogenesis and activates antioxidant enzymes, enhances chemoresistance in response to ROS generated by exposure of cells to ovarian sphere-forming culture conditions. Cells in the cultured spheres exhibited stem cell-like characteristics, and maintained higher ROS levels than their parent cells. Intriguingly, scavenging ROS diminished the aldehyde dehydrogenase (ALDH)-positive cell population, and inhibited proliferation of the spheres. ROS production triggered PGC1α expression, which in turn caused changes to mitochondrial biogenesis and activity within the spheres. The drug-resistant phenotype was observed in both spheres and PGC1α-overexpressing parent cells, and conversely, PGC1α knockdown sensitized the spheres to cisplatin treatment. Similarly, floating malignant cells derived from patient ascitic fluid included an ALDH-positive population and exhibited the tendency of a positive correlation between expressions of multidrug resistance protein 1 (MDR1) and PGC1α. The present study suggests that ROS-induced PGC1α mediates chemoresistance, and represents a novel therapeutic target to overcome chemoresistance in ovarian cancer.

## INTRODUCTION

Tumors undergo metabolic reprogramming to meet the increased energetic and anabolic demands [1]. A number of previous reports have shown that according to the Warburg effect, cancer cells typically rely on aerobic glycolysis due to exhibited defects in mitochondrial function [2, 3]. Tumor cells, however, still possess a capacity to synthesize significant amounts of triphosphate (ATP) if challenged by glucose depletion, via the mitochondrial oxidation of fatty acids and amino acids. This cellular production of ATP and the corresponding intermediate metabolites is a major function of mitochondria. The TCA intermediates and the reducing coenzymes, such as NADH and FADH_2_, contribute to the production of macromolecules as building blocks for cancer cells [4]. In fact, cancer cells exhibit highly functional mitochondria that efficiently produce both energy and anabolic materials.

Reactive oxygen species (ROS) are byproducts of mitochondrial metabolism, and function as second messengers in the transduction of extracellular signals that control cellular proliferation and cell cycle progression. The altered metabolism exhibited by tumor cells causes ROS production and sustains ROS at an aberrantly high level [5], thereby driving cancer cells to develop an adaptive system against oxidative stress [6]. In one such detoxifying mechanism, PGC1α, as a major regulator of mitochondrial biogenesis and respiratory function, is required for induction of ROS-detoxifying enzymes, and also contributes to the reduction of ROS generation by mitochondrial metabolism [7–9]. Under the normal conditions, PGC1α is sustained at low levels, but its expression is elevated in response to increasing bioenergetic demands and metabolic alterations [8]. Recently, PGC1α was reported to exhibit oncogenic properties in some cancer cells [10–13]. However, the effect of PGC1α on chemoresistance in ovarian cancer has not yet been elucidated.

Ovarian cancer is usually diagnosed at advanced stages due to difficulty in early detection [14]. More than one third of patients with ovarian cancer suffer from malignant ascites, which is a condition considered to mark the development of chemoresistance and metastasis, resulting in poor prognosis [15–18]. Ascites is composed of acellular fluid containing soluble factors and heterogenic cellular components as a form of single cells or more commonly aggregates and spheres [19]. Floating condition, either as aggregates or spheres, in the ascitic fluid helps cells to survive in an anchorage-independent environment and protects them against penetration of chemotherapeutic agents [20]. In addition, a proportion of malignant cells forming aggregates or spheres have features of cancer stem cell (CSC)-like phenotypes contributing to drug resistance and metastasis [20, 21]. *In vitro* models in which a non-adherent culture condition mimics the ascitic environment can be used to effectively investigate advanced ovarian cancer, and identify novel therapeutic targets. In the present study, we attempted to identify biological changes associated with chemoresistance using a non-adherent culture consisting of multicellular spheres with CSC-like phenotypes. Using this sphere cell culture, we demonstrated that PGC1α induced by ROS generation facilitates mitochondrial biogenesis and attenuates mitochondrial activity to confer chemoresistance to ovarian cancer cells.

## RESULTS

### Sphere formation increases the CSC population and displays enhanced drug resistance

**Figure 1:**
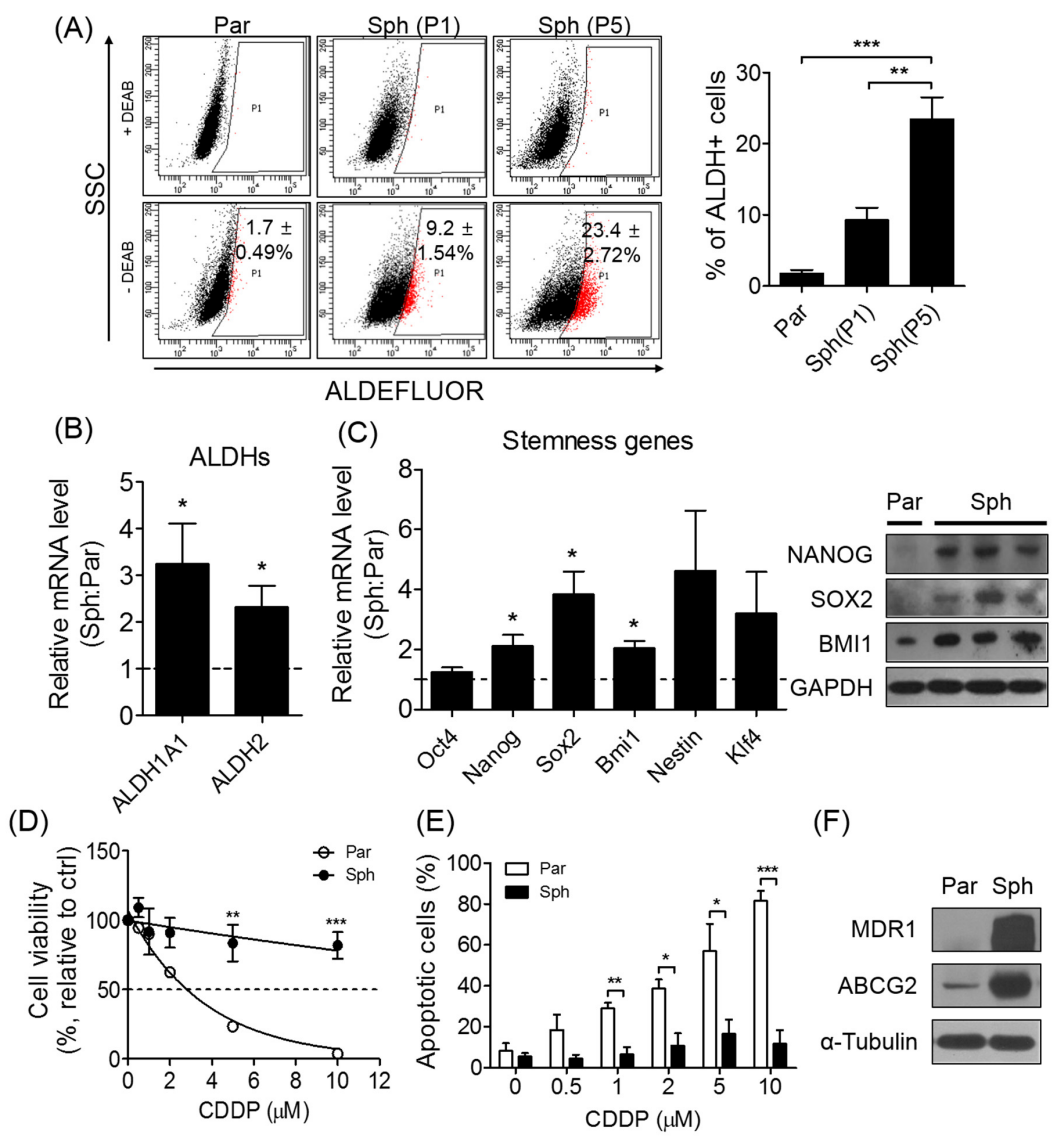
Sphere formation enriches stem-like population and exhibits drug-resistance in ovarian cancer cells sensitive to CDDP **(A)** ALDH activity of parent cells and spheres at passage 1 (P1) and 5 (P5) was detected by ALDEFLUOR (par, parent cells; sph, spheres). **(B, C)** Sphere formation increased mRNA expression of *ALDHs* and stemness-related genes (*Oct4, Nanog, Sox2, Bmi1, Nestin,* and *Klf4*). Relative mRNA expression was evaluated by quantitative real-time PCR (qRT-PCR) and normalized to parent cells. NANOG, SOX2, and BMI1 protein were expressed in spheres. **(D, E)** The indicated concentrations of CDDP were treated to spheres and parent cells for 48 h. Cell viability was determined by MTT assay. Apoptotic population was determined by flow cytometry following annexin-V and propidium iodide staining. **(F)** Expression of drug resistance-related proteins, MDR1 and ABCG2, was detected by Western blot. All data were presented as the mean ± SEM of independent experiments (N = 5; *p < 0.05, **p < 0.01, ***p < 0.001, spheres vs. parent; ANOVA with Scheffe's post hoc test in 1A; Student's t-test in 1B, 1C, 1D, and 1E).

Although PA1 is an ovarian cancer cell line derived from the ascites of a patient with teratocarcinoma, we selected PA1 sensitive to a platinum-based chemotherapy to examine whether sphere-forming culture conditions induce chemoresistance. As expected, sphere-culture conditions resulted in an enriched CSC population with a high ALDH activity. Compared to the parent cells (attached/two dimensional-cultured), the ALDH activity exhibited by the CSCs in ovarian tumor spheres was significantly increased (Figure 1A). Serial subculturing of the spheres (passage 1 and 5) enriched the ALDH-positive population (Figure 1A), and mRNA expression for two subtypes of ALDH and stemness-related genes including *Nanog*, *Sox2*, and *Bmi1* was also increased in spheres relative to parent cells (Figure 1B and 1C). To confirm the resistance of spheres to a platinum-based chemotherapeutic agent, cisplatin (CDDP), we assessed the effect of treating both parent cells and spheres with the serial concentrations of CDDP or paclitaxel, and found that the spheres exhibited a higher IC_50_ than their parent cells (Figure 1D, Supplementary Figure 2A). The number of apoptotic cells was found to be significantly decreased (Figure 1E), while conversely, the expression of drug-resistance-related MDR1 and ABCG2 proteins (Figure 1F) was significantly increased in spheres. Taken together, these results suggest that sphere formation enriches the population of stem-like cells in the PA1, and thereby confers drug-resistance.

### ROS generated by sphere formation are related to the stem-like phenotype of ovarian cancer cells

**Figure 2:**
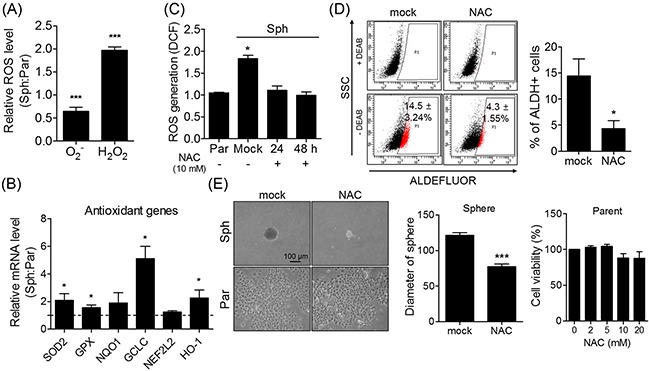
Sphere formation causes an increase of ROS level accompanying stem-like phenotypical changes **(A)** H_2_O_2_ and O_2_^−^ levels of spheres and parent cells were detected using flow cytometry after DCFH-DA and DHE staining, respectively. Relative H_2_O_2_ and O_2_^−^ levels of spheres were presented after normalized to parent cells. **(B)** Spheres showed higher expression of several antioxidant genes relative to parent cells. The relative mRNA expression was determined by qRT-PCR. **(C)** NAC treatment (10 mM) decreased the ROS content produced in spheres. **(D)** ALDH-positive population was detected in NAC-treated and non-treated spheres (10 mM for 4 days). Treating NAC alleviated ALDH activity in spheres. ALDEFLUOR assay kit was used to assess a proportion of ALDH activity. **(E)** Spheres and parent cells were cultured with/without 10 mM NAC for 4 days. The morphology of spheres and parent cells was determined by phase-contrast microscopy (Scale bar, 100 μm). Arbitrary unit was used for measuring the diameter of NAC-treated and non-treated spheres using ImageJ software. All data were presented as the mean ± SEM of independent experiments (N = 5; *p < 0.05, ***p < 0.001; Student's *t*-test).

Sphere formation has been previously shown to stimulate ROS generation [22]. In the present study, hydrogen peroxide (H_2_O_2_) and superoxide (O_2_^−^) were increased and decreased, respectively, in the spheres compared to their parent cells (Figure 2A). Furthermore, the spheres exhibited relatively high antioxidant gene expression levels in response to endogenous ROS (Figure 2B), while N-acetyl-cisteine (NAC, ROS scavenger) treatment decreased ROS level produced in spheres, and reduced the ALDH activity increased in spheres (Figure 2C and 2D). NAC treatment also decreased the size of the spheres, but did not affect the morphology nor viability of parent cells (Figure 2E). These findings indicate that the intracellular ROS generation caused by sphere formation induces phenotypical changes in CSCs.

### Sphere formation induces PGC1α expression and alters mitochondrial dynamics, biogenesis, and activity

**Figure 3:**
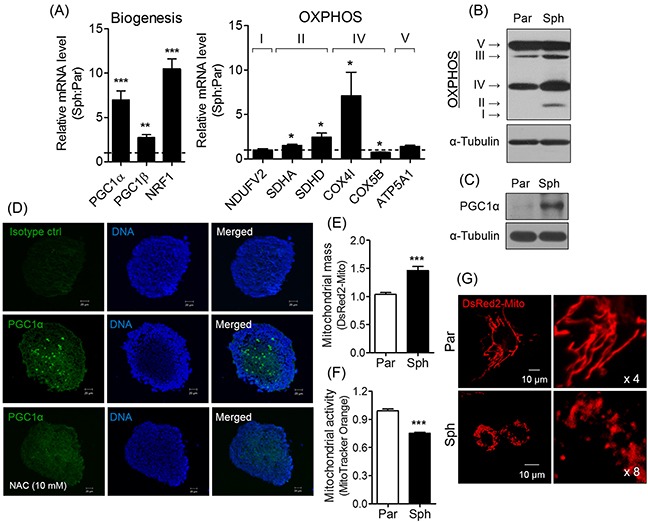
Sphere formation induces PGC1α expression, resulting in mitochondrial changes **(A)** Relative mRNA expression of genes related to mitochondrial biogenesis and OXPHOS was increased in spheres. Values were normalized to parent cells. **(B)** Increased expression of OXPHOS was observed in spheres (par, parent cells; sph, spheres). **(C)** PGC1α expression was verified by Western blotting. **(D)** PGC1α expression in the spheres and NAC-treated spheres was detected by immunocytochemistry. Blue and green signals are DAPI and PGC1α, respectively. Isotype IgG was used as a control. **(E)** pDsRed2-Mito vector was introduced to parent and spheres to determine mitochondrial mass. Mitochondria labeled with red fluorescence was detected by flow cytometry. **(F)** MitoTracker Orange was stained to parents and spheres to confirm mitochondrial activity, and detected by flow cytometry. **(G)** Parents showed tubular structure, while spheres showed fragmented mitochondria. Mitochondrial morphology was observed using an LSM confocal microscope following fluorescent labeling of mitochondria using pDsRed2-Mito vector. All data were presented as the mean ± SEM of independent experiments (N = 3; *p < 0.05, **p < 0.01, ***p < 0.001; Student's *t*-test).

To confirm that the ROS-induced PGC1α expression promotes cell detoxification, we analyzed the expression of genes related to mitochondrial biogenesis (PGC1α, −1β, and NRF1) and oxidative phosphorylation (OXPHOS; SDHA, SDHD, and COX4I; Figure 3A). Among the genes considered, we focused on PGC1α involved in mitochondrial biogenesis and metabolism [23]. The expressions of OXPHOS complex II, III, and IV, and PGC1α (Figure 3B and 3C) was enhanced by sphere formation, and PGC1α expression was observed to be localized near the center of the spheres (Figure 3D). In addition, sphere formation elevated the mitochondrial mass, and reduced mitochondrial activity (Figure 3E and 3F), which is influenced by mitochondrial dynamics (i.e. fusion and fission) [24]. As predicted, sphere formation altered mitochondrial structure. In contrast to parent cells exhibited elongated mitochondria, the spheres were observed to be markedly fragmented at the perinuclear region (Figure 3G). These findings suggest that sphere-induced ROS generation increases PGC1α expression and mitochondrial biogenesis, but reduces mitochondrial activity via induced mitochondrial fission.

### ROS-induced PGC1α mediates chemoresistance of ovarian cancer cells

**Figure 4:**
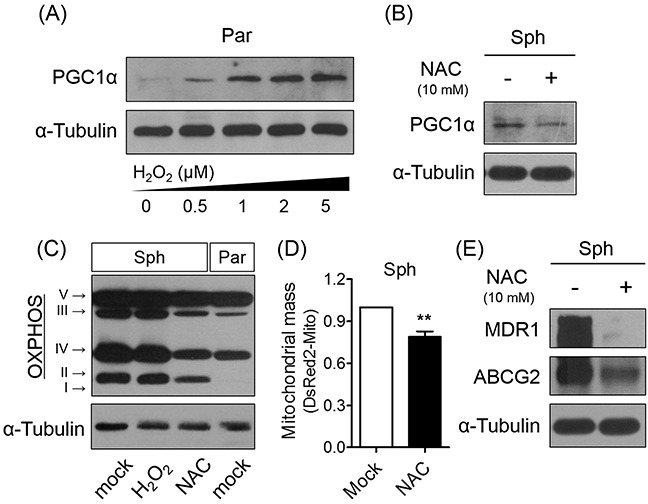
Scavenging ROS decreases PGC1α and OXPHOS expression **(A)** Exogenous addition of ROS caused PGC1α expression in parent cells. **(B, C)** Inhibition of ROS generation using NAC (10 mM for 4 days) showed down-regulation of PGC1α and OXPHOS expression in spheres. **(D)** Scavenging ROS decreased mitochondrial mass labeled with pDsRed2-Mito in spheres. **(E)** Scavenging ROS in spheres down-regulated expression of MDR1 and ABCG2 proteins (N = 3; **p < 0.01, mock vs. NAC treatment, Student's *t*-test in 4D).

PGC1α as a signaling molecule regulates the expression of antioxidant enzymes [7]. To confirm that PGC1α expression within the spheres is induced by ROS generation, including particularly H_2_O_2_, we assessed the effect of treating parent cells with various concentrations of H_2_O_2_. The results of the analysis showed that increased exogenous H_2_O_2_ levels correlated with increased PGC1α expression (Figure 4A). However, exogenous H_2_O_2_ addition to the spheres did not increase PGC1α, MDR1, and OXPHOS protein levels (Supplementary Figure 1). Conversely, ROS elimination via NAC treatment caused a reduction in PGC1α and OXPHOS complex expression in spheres, as well as a corresponding decrease in mitochondrial mass (Figure 4B, and 4D). Interestingly, inhibiting ROS generation induced down-regulation of MDR1 and ABCG2 in spheres (Figure 4E). Taken together, these results suggest that ROS-mediated PGC1α induction results in mitochondrial biogenesis and structural changes, and scavenging ROS sensitizes the spheres to CDDP treatment via down-regulation of PGC1α and associated drug resistance-related proteins.

### PGC1α protects spheres against CDDP treatment

**Figure 5:**
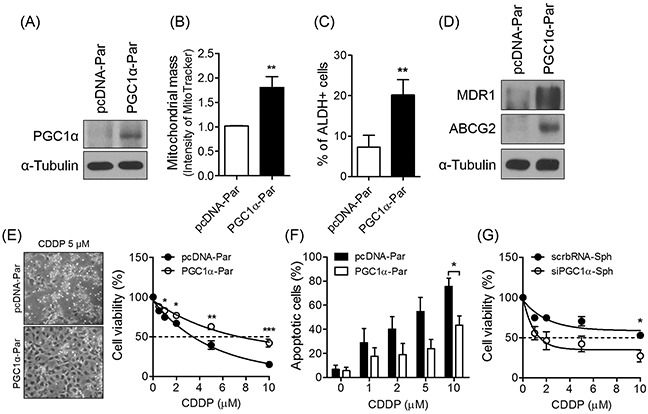
PGC1α is involved in the enhanced chemoresistance **(A)** Parents were transfected with pcDNA-PGC1α (PGC1α-par) and pcDNA (pcDNA-par). **(B)** Overexpressed PGC1α in parent cells increased mitochondrial mass. Mitochondrial mass of PGC1α-par and pcDNA-pars was analyzed by flow cytometry after stained with 300 nM MitoTracker Green FM dye. **(C)** PGC1α-par showed increased ALDH-positive population. **(D)** Overexpression of PGC1α in parents enhanced the expression of drug resistance-related proteins. **(E)** PGC1α-par and pcDNA-par were treated with various concentrations of CDDP for 48 h. The morphological difference and viability were determined by a phase-contrast microscope and MTT assay, respectively. **(F)** Apoptosis was decreased in PGC1α-par. **(G)** Silencing of PGC1α sensitized spheres to cell death by CDDP. Small interference RNA (50 nM) specific for PGC1α (siPGC1α) and scrambled RNA (scrbRNA) were transfected to sphere-dissociated cells, and the cells were cultured one week for re-forming spheres. CDDP was treated to siPGC1α and scrbRNA-transfected spheres for 48 h. Data were presented as the mean ± SEM of independent experiments (N = 3; *p < 0.05, **p < 0.01, ***p < 0.001, Student's *t*-test).

To determine whether expression of PGC1α is correlated with drug-resistance, we evaluated the effect of overexpressing PGC1α in parent cells. We conducted an immunoblot analysis to confirm that PGC1α was overexpressed following pcDNA-PGC1α transfection in parent cells (Figure 5A). Expectedly, PGC1α-overexpression in parent cells caused an increase in mitochondrial mass to a level comparable to that exhibited by the spheres (Figure 5B). We next further investigated the effect of PGC1α overexpression on the acquisition of CSC-like phenotypes by conducting an ALDEFLUOR assay, and verified conferred drug-resistance via an MTT assay and annexin V-PI staining. The results of these analyses showed that compared to parent cells, PGC1α overexpression increased both the ALDH activity and expression of drug-resistant proteins in spheres (Figure 5C and 5D). To evaluate drug resistance, we treated parent cells and PGC1α-overexpressing cells with various concentrations of CDDP or paclitaxel for 48 h. The PGC1α-overexpressing cells exhibited a higher IC_50_ value of CDDP or paclitaxel than parent cells (Figure 5E, Supplementary Figure 2C), and the number of apoptotic cells was also significantly lower in the PGC1α-overexpressing cells than in parent cells following CDDP treatment (Figure 5F). In contrast, PGC1α silencing sensitized spheres to CDDP or paclitaxel treatment (Figure 5G, Supplementary Figure 2B). Thus, these results indicate that PGC1α mediates chemoresistance in ovarian cancer cells.

### PGC1α is partially involved in chemoresistance of ascites-derived cancer cells from ovarian cancer patients at the advanced stage

**Figure 6:**
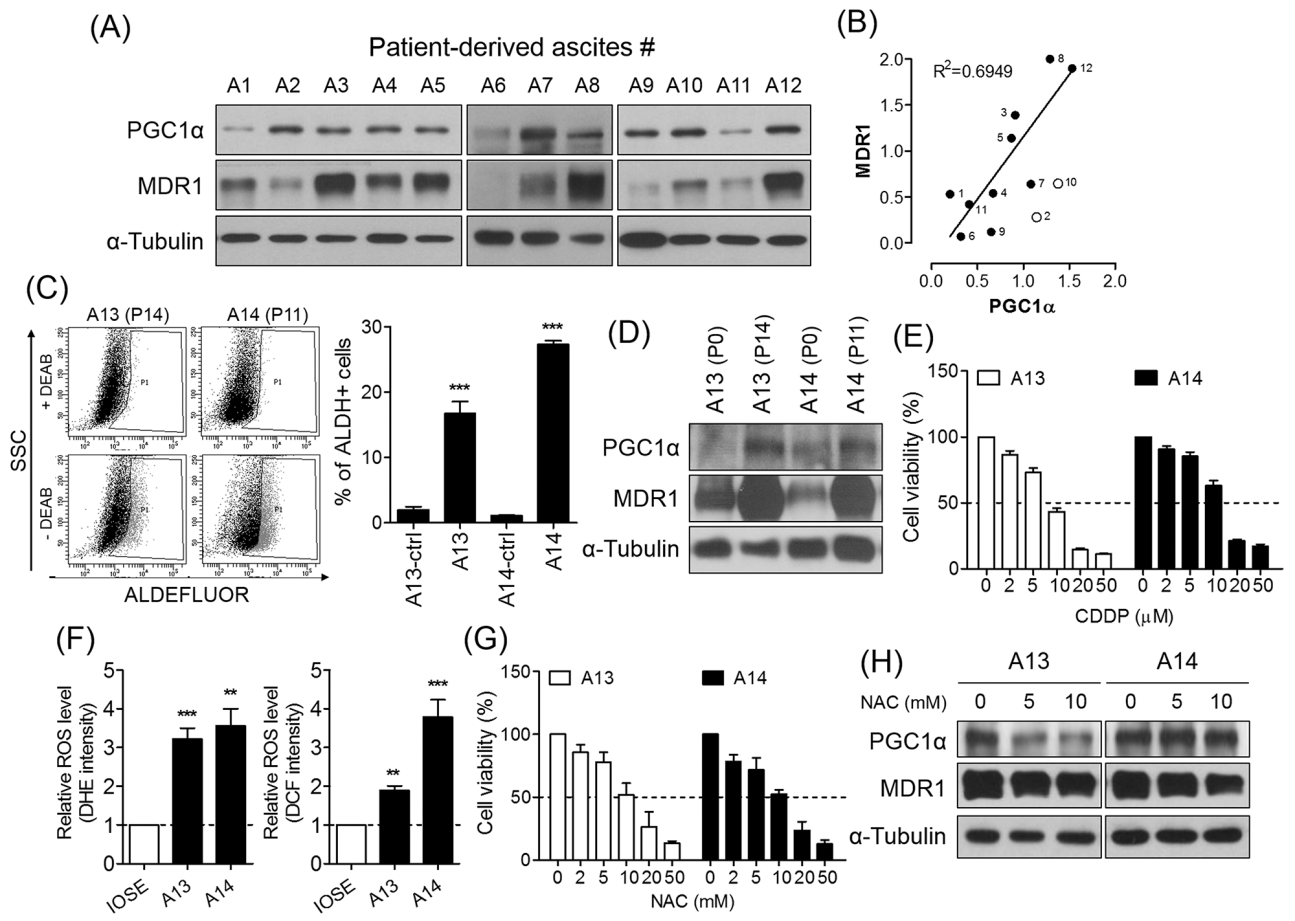
PGC1α is partially involved in chemoresistance of ovarian cancer patient-derived ascites cells **(A)** Ascites-derived cells collected from ovarian cancer patients expressed PGC1α and MDR1. Protein levels were determined by Western blot. **(B)** Positive correlation between PGC1α and MDR1 was shown in 12 samples of ascites (p = 0.0027). **(C, D)** ALDH activity of A13 at passage 14 (P14) and A14 at P11 was detected by ALDEFLUOR. Serial subculture of ascites-derived cells increased ALDH-positive population and expression of PGC1α and MDR1. **(E)** Viability of ascites-derived cancer cells was determined by MTT. **(F)** Intracellular generation of superoxide anion and hydrogen peroxide was detected by DHE and DCFDA in ascites-derived cancer cells. IOSE was used as control. **(G)** After treatment of serial concentrations of NAC for 48 h, viability of ascites-derived cancer cells was determined by MTT assay. **(H)** In ascites-derived cancer cells treated with NAC for 48 h, PGC1α and MDR1 expression were measured by Western blot. All data were presented as the mean ± SEM of independent experiments (N = 3; **p < 0.01, ***p < 0.001, ctrl or IOSE vs. ascites-derived cells, Student's *t*-test).

Malignant cells are present in ascites of patients with ovarian cancer, either as single cells, or aggregates (Supplementary Figure 3) [17]. Recent studies have demonstrated that a small proportion of the total ascites cells expresses pluripotent genes and putative CSC markers such as ALDH enzymatic activity [25, 26]. Therefore, we hypothesized that putative CSCs in ascites may be associated with PGC1α-mediated chemoresistance. To test the relationship between PGC1α and chemoresistance in patient-derived cancer cells, we collected and isolated the cellular portion of 14 ascites from patients with serous, clear cell, and endometrioid ovarian cancer at advanced stage III – IV (Table 1). Of 12 collected patient samples, the majority of ascites-derived cells expressing PGC1α displayed MDR1 expression (Figure 6A), such that we identified a positive correlation between PGC1α and MDR1 expression that was independent of histologic subtypes (p = 0.0027; Figure 6B). To enrich cancer cells from heterogenic ascites-derived cells, we performed serial subcultures of ascites 13 and 14 which were classified as being serous and clear cell carcinoma subtype, respectively. Two enriched cancer cells (A13 at passage 14 and A14 at passage 11) had a large ALDH-positive population as much as PA1 spheres (Figures 1A and 6C). We found that ascites-derived cancer cells with ALDH-positive population expressed high levels of PGC1α and MDR1 (Figure 6D), and were relatively resistant to CDDP treatment compared to PA1 parent cells (Figures 1D and 6E). When we assessed the relationship between chemoresistance and ROS production, we observed that superoxide anion and hydrogen peroxide generation was significantly increased in the ascites-derived cancer cells compared to immortalized normal ovarian surface epithelial cells (IOSE; Figure 6F). Reduction of ROS levels via NAC treatment decreased the viability of the ascites-derived cancer cells (Figure 6G), and inhibited their expression of PGC1α and MDR1 (Figure 6H). These findings suggest that, similar to the results generated during analysis of the spheres, ROS-induced PGC1α mediates the chemoresistance of ascites-derived cancer cells with ALDH-positive population.

**Table 1:** Information of ovarian cancer patients with ascites

Ascites No.	Histological type	Stage
A1	Serous	IIIC
A2	Serous	IIIC
A3	Clear cell	IVB
A4	Endometrioid	IV
A5	Serous	IVB
A6	Serous	IV
A7	Clear cell	III
A8	Serous	IIIC
A9	Serous	IVA
A10	Serous	IV
A11	Serous	IVB
A12	Serous	IIIC
A13	Serous	IIIC
A14	Clear cell	IIIC

## DISCUSSION

To date, it has been generally thought that cancer cells dominantly utilize glycolysis to meet the metabolic demands, due to an increased incidence of mitochondrial defects [3]. However, emerging evidence reveals that cancer cells have functional mitochondria that mediate tumorigenesis. In the present study, we provide the evidence that ROS-induced PGC1α mediates chemoresistance in ovarian cancer cells. Currently, platinum-based therapy is the standard treatment used for ovarian cancer. We observed that PA1 cells sensitive to CDDP acquired drug resistance when they were stimulated by exposure to specific culture conditions to form spheres, because this process stimulated ROS generation, and induced the expression of detoxifying enzymes and PGC1α. In turn, PGC1α induction in the spheres caused mitochondrial biogenesis and structural changes, and enhanced drug resistance by mitigating oxidative stress induced by ROS-inducing drugs. However, exogenous H_2_O_2_ addition to the spheres after 3D-structure formation did not increase PGC1α and OXPHOS protein levels in a H_2_O_2_ concentration-dependent manner compared to mock-treated spheres, because PGC1α already activated inside the spheres during 3D-structure formation.

Exposure to culture conditions conductive to sphere formation is considered as an efficient method for the enrichment and isolation of CSCs [27–30]. In addition, three-dimensional (3D) culture via sphere formation is a representative method used to mimic *in vivo* tumor microenvironment. Because of limitation of oxygen penetration, the inside of *in vitro*-cultured spheres (3D culture) is in both oxidative stress and hypoxic conditions, which activate antioxidant proteins and HIF contributing to resistance to chemotherapy [22, 31–33]. In advanced ovarian cancers, malignant cells floating in ascites as a form of single cells, aggregates and/or sphere have the ability to metastasis to distant sites and to protect themselves against chemotherapy [15, 19]. It has been previously proposed that malignant cells with cancer stem-like phenotypes are enriched in aggregates and/or spheres within ascites. In fact, ascites-derived spheres expressing Oct4, STAT3, and CA125 have been shown to exhibit a strong tumorigenic ability in nude mice compared to single cells [20]. According to the results of the present study, the microenvironmental condition of spheres mimics that of an *in vivo* tumor mass by enabling ovarian cancer cells to acquire stem-cell properties, including high ALDH activity and drug resistance. Interestingly, 2D and 3D culture conditions facilitated opposite cell responses to NAC treatment. In the patient-derived samples, the expressions of MDR1 was positively correlated with that of PGC1α in ascites cells. This implies that the physical tumor microenvironment should be considered a factor that critically influences cancer-cell phenotypes.

While antioxidants are generally established to exert a protective effect against carcinogenesis by reducing DNA damage, recent research revealed that ROS scavengers, including NAC, inhibit the growth and proliferation of glioblastoma-initiating cells via the impairment of cell cycle progression [34]. In addition, it was reported that elevated ROS produced by prostate tumor initiating cells was required for the activation of IL-6/STAT3, which was related with carcinogenesis of human prostate cells [33]. Intracellular ROS as second messenger stimulate cell proliferation and regulate [35]. The results of the present study demonstrated that the utilized 3D culture condition facilitates ROS generation, and thus enables ROS to stimulate proliferation of tumor cells. A reduction in the level of ROS decreases the size and the number of incident spheres, concomitantly with a reduction in ALDH activity. In addition, up-regulation of antioxidant gene expression is observed in spheres accompanying ROS elevation. We observed a decreased level of superoxide and an increased level of hydrogen peroxide, because superoxide dismutase 2 (SOD2) may act in the process to convert superoxide to hydrogen peroxide in mitochondria. Similarly, accumulating evidence suggests that SOD2 expression is correlated with chemoresistance in lymphoma [36], basal-like breast carcinoma [37], and lung adenocarcinoma [38]. In line with previous data, the results of the present study suggest that acquisition of chemoresistance and stem-like phenotypes in spheres is likely associated with the ROS-induced expression of detoxifying enzymes. Thus, removal of ROS may be a promising strategy to target proliferative CSCs in ovarian cancer.

PGC1α critically regulates the transcriptional control of mitochondrial metabolism and biogenesis [9, 23]. Recent studies have suggested that expression of PGC1 family members is associated with oncogenic processes, including metastasis and chemoresistance of cancers. PGC1α and mitochondrial transcription factor A (TFAM) were found to be increased in high grade serous ovarian cancers that were highly chemoresistant [39]. Similarly, circulating breast cancer cells have been found to exhibit enhanced mitochondrial biogenesis and respiration as a result of induced PGC1α expression, leading to an increased rate of metastasis [11]. An increased reliance on OXPHOS with a marked shift away from aerobic glycolysis was observed in glioma stem cells [40]. Similarly, high PGC1α and HIF1α levels have been suggested as effective and non-invasive plasma biomarkers of poor prognosis for patient with breast cancer [41]. The findings of the present study support a role for PGC1α in the regulation of oncogenic properties. Sphere formation-induced ROS generation was shown to stimulate PGC1α expression, leading to the increased expression of OXPHOS-related genes and proteins. Although mitochondrial fission was observed in spheres, mitochondrial mass was also increased to compensate for the inefficient energy metabolism caused by mitochondrial fission under low oxygen conditions.

Thus, the present study provides evidence that PGC1α induced by oxidative stress mediates chemoresistance in ovarian cancer. Many previous studies have focused on the increase of mitochondria that occurs in relation to caspase-dependent death pathways in cancer; however, the findings of the present study demonstrate the contradictory functions PGC1α-regulated mitochondria in mediating cancer cell survival. Although further studies are required to elucidate the molecular mechanisms underlying our findings, we propose that PGC1α is a key regulator that controls chemoresistant phenotypes in ovarian cancers.

## MATERIALS AND METHODS

### Culture of ovarian cancer cells

Ovarian cancer cell line, PA1 was purchased from American Type Culture Collection, and cultured in DMEM/F12 (Life Technologies, Gaithersburg, MD) supplemented with 10% fetal bovine serum (FBS; Life Technologies), 1% penicillin and streptomycin (Life Technologies). PA1 cells were maintained at 37°C in humidified atmosphere of 5% CO_2_.

### Tumor sphere formation

PA1 cells (1,000 cells/cm^2^) were plated onto poly-2-hydroxyethyl methacrylate (10 mg/ml, poly-HEMA; Sigma Aldrich, St. Louis, MO) coated plate, and cultured in DMEM/F12 containing 20 ng/ml human recombinant epidermal growth factor (EGF; Life Technologies), 20 ng/ml basic fibroblast growth factor (bFGF; Life Technologies), 5 μg/ml human recombinant insulin (Life Technologies), 0.1x B27 (Life Technologies), and 1% penicillin and streptomycin (Life Technologies). Spheres were maintained at 37°C in humidified atmosphere of 5% CO_2_ for two weeks.

### Measurement of ALDH activity

Cancer stem-like cells with high ALDH activity were identified in spheres and parent cells (1 × 10^6^ cells) using ALDEFLUOR® assay kit (STEMCELL Technologies, Vancouver, Canada). The spheres were dissociated with 0.05% trypsin-EDTA at 37°C for 2 min. Trypsin-EDTA treated spheres were centrifuged at 4°C to avoid further enzymatic damage (700 × g, 10 min). Dissociated spheres and parent cells (each 1 × 10^6^ cells) were suspended in ALDEFLUOR assay buffer containing ALDH substrate without/with 50 mM DEAB (as a negative control, specific inhibitor to ALDH), and incubated for 30 min at 37°C. ALDH-positivity was analyzed by BD FACSCanto II flow cytometer (BD Biosciences, NorthRyde, Australia).

### Measurement of intracellular ROS levels in ovarian cancer cells

ROS level of spheres and parental cells was measured by dihydroethidium (DHE; Sigma Aldrich, St. Louis, MO) and 6-carboxy-2,7-dichlorodihydrofluorescein diacetate (DCFH-DA; Sigma Aldrich) to detect superoxide anion and hydrogen peroxide, respectively. The spheres and parental cells were dissociated with 0.05% trypsin-EDTA at 37°C for 2 min. Trypsin-EDTA treated spheres were centrifuged at 4°C to avoid further enzymatic damage (700 × g, 10 min). The dissociated parental cells were centrifuged at 500 × g, 4°C for 4 min. Both dissociated spheres and parental cells were incubated in serum-free medium with DHE (5 μM for 10 min) or DCFH-DA (20 μM for 30 min) at 37°C in the dark. Relative fluorescence intensity of ethidium (ETH, oxidized form of DHE by superoxide) or DCF (oxidized form of DCFH by hydrogen peroxide) was measured by BD FACSCanto II flow cytometer (BD Biosciences).

### Total RNA isolation and quantitative real time-PCR (qRT-PCR)

Total RNA was extracted from spheres and parent cells with TRIzol reagent (Invitrogen). Complementary DNA (cDNA) was synthesized from 1μg of total RNA using superscript III first-strand synthesis system with oligo(dT)_20_ primers (Invitrogen). The synthesized cDNA was diluted with nuclease free water (five-fold), and mixed with Master Mix SYBR Green I dye (Bio-Rad, Hercules, CA). Relative gene expression levels were measured by CFX96 Real-Time PCR Detection System (Bio-Rad) according to the manufacturer's protocol. Amplification conditions were followed for 35 cycles at 94°C for 30 s, 60°C for 30 s, and 72°C for 30 s. The relative gene expression levels were quantified using the 2^−ΔΔCt^method and normalized to the C_t_ value of the reference genes, β-actin and GAPDH.

### Western blotting

Spheres and parent cells were harvested and lysed with protein extraction buffer (0.5 M NaCl, 0.5 M Tris-HCl, 50 mM EDTA, 50 mM EGTA, 10% triton X-100, 1 mg sodium deoxycholate, 1 mM Na_3_VO_4_, 1mM phenylmethylsulfonyl fluoride, EDTA-free protease inhibitor, and distilled water). Proteins (20 μg) were separated on 6 - 10% SDS-PAGE, and transferred onto a nitrocellular membrane. After blocked with 5% skim milk solution in Tris-buffered saline with 0.1% Tween 20 (0.1% TBS-T), the membranes were incubated with primary antibodies specific for MDR1 (Cell Signaling Technology, Beverly, MA), ABCG2 (Santa Cruz Biotechnology, Santa Cruz, CA), total OXPHOS complexes (Abcam, Cambridge, MA), and PGC1α (Calbiochem, Darmstadt, Germany). Signals were visualized using a chemiluminescence detection kit (AbFrontier, Seoul, Korea).

### MTT assay

To measure cell viability after treated with cisplatin (CDDP), cultured cells (spheres and parent cells) were incubated with 2 mg/ml of 3-(4,5-dimethylthiazol-2-yl)-2,5-diphenyltetrazolium bromide (MTT) dissolved in phosphate buffered saline (PBS) solution at 37°C in the dark. After incubation for 3 h, MTT was removed, and dimethyl sulfoxide (DMSO) was added to dissolve formazan in the live cells. Aliquots (200 μl) of DMSO solution were transferred into 96-well plates, and absorbance was recorded at 540 nm using a spectrophotometer (Labsystem Multiskan, Helsinki, Finland)

### Measurement of apoptosis

Spheres were cultured for two weeks and treated with various concentrations of CDDP for 48 h. Drug-treated cells were dissociated with 0.05% Trypsin-EDTA, and centrifuged at 500 × g for 4 min. The cell pellet was suspended in PBS and stained using annexin V-PI apoptosis detection kit (BD Bioscience Pharmingen, San Jose, CA). Apoptotic cells were analyzed by BD FACSCanto II flow cytometer using the CellQuest analysis program (BD Biosciences, NorthRyde, Australia).

### Measurement of mitochondrial activity

To measure the relative mitochondrial activity compared to parent cells, both parent cells and spheres were dissociated and incubated in serum-free medium containing 200 nM MitoTracker® Orange CMTMRos (Invitrogen) at 37°C for 30 min. After incubation, the stained cells were centrifuged at 500 × g for 4 min, and washed with PBS. The cells were analyzed by BD FACSCanto II flow cytometer (BD Bioscience).

### Immunocytochemistry for the detection of PGC1α in spheres

After culture for two weeks, sphere samples were fixed with 4% formaldehyde at 4°C overnight. Fixed samples were washed with PBS for 30 min (three times every 10 min) followed by dehydration through a graded ethanol of 25, 50, 70, 90, and 100% for 10 min in each step. Paraffin-embedded preparations of sphere samples were sectioned at 7 μm thickness by a microtome (HM340E; Microm, Waldorf, Germany). Water drops were put on VWR® Micro Slides (VWR International, West Chester PA), and the sectioned paraffin-embedded samples were put on the water drops to help the samples to attach to the slides. The sections on the slides were dried at 40°C overnight to vaporize water drops, and dewaxed with CitriSolv (Fisher BioSciences, Pittsburgh, PA). After rehydrated with a graded ethanol of 100, 70, 50, and 25%, and PBS for 10 min in each step, samples were blocked in PBS containing 0.1% Tween-20 (0.1% PBS-T) and 3% normal goat serum (Vector Laboratories, Burlingame, CA) at room temperature for 1 h.

Sectioned samples were incubated in 0.1% PBS-T containing 3% normal goat serum and primary antibody (anti-PGC1α mouse antibody, 1:200; anti-ABCG2 mouse antibody, 1:500) at 4°C overnight. After washed in 0.1% PBS-T for 60 min (three times every 20 min), samples were incubated in 0.1% PBS-T containing secondary antibody (anti-mouse IgG conjugated with AlexaFluor488, 1:250) for 4°C overnight. Samples were washed with 0.1% PBS-T for 60 min (three times every 20 min), and mounted with mounting medium (Vectashield with DAPI, H-1200; Vector Laboratories). Fluorescent images were captured using LSM 700 confocal microscope (Zeiss, Germany).

### Overexpression of PGC1α

PA1 parent cells were transfected with pcDNA4-myc-PGC1α using Lipofectamine® 2000 transfection reagent (Life Technologies). The pcDNA4-myc-PGC1α was a gift from Toren Finkel (Addgene plasmid # 10974) [42]. Transfected cells were selected with 400 μg/ml Zeocin (Invitrogen) during two weeks.

### Silencing of PGC1α

Small interfering RNA (siRNA) was obtained from Bioneer (Daejeon, Korea). Spheres were dissociated and transfected with siRNA against PGC1α (Sense: CAAUAACUCCACCAAGAAA, Antisense: UUUCUUGGUGGAGUUAUUG) and scrambled siRNA (negative control) using Lipofectamine® 2000 transfection reagent (Life Technologies). The transfected cells were cultured onto a poly-HEMA coated plate for one week.

### Isolation of ascites cells from ovarian cancer patients with ascites

Ascites was aspirated from ovarian cancer patients with different histological types including serous, clear cell, and endometrioid type, at the stage III and IV. Ascites was diluted with the same volume of PBS, and centrifuged (4°C, 1,400 × g, 10 min). Cells were gently overlaid onto Ficoll-Paque^TM^-PREMIUM, and centrifuged (4°C, 1,400 × g, 30 min). The cell layer was collected and cultured in the complete culture medium.

### Statistical analysis

Data were presented as mean ± SEM of at least three independent experiments. Student's *t*-test and one-way ANOVA were used for statistical analyses. Significant difference among experimental groups was analyzed by Scheffe's post hoc test. All analyses were conducted using IBM SPSS statistics 23 (SPSS Inc., Chicago, IL).

## SUPPLEMENTARY MATERIALS FIGURES AND TABLES



## References

[1] Ward PS, Thompson CB (2012). Metabolic reprogramming: a cancer hallmark even warburg did not anticipate. Cancer Cell.

[2] Vander Heiden MG, Cantley LC, Thompson CB (2009). Understanding the Warburg effect: the metabolic requirements of cell proliferation. Science.

[3] Warburg O (1956). [Origin of cancer cells] [Article in German]. Oncologia.

[4] Weinberg SE, Chandel NS (2015). Targeting mitochondria metabolism for cancer therapy. Nat Chem Biol.

[5] Weinberg F, Chandel NS (2009). Mitochondrial metabolism and cancer. Ann N Y Acad Sci.

[6] Sabharwal SS, Schumacker PT (2014). Mitochondrial ROS in cancer: initiators, amplifiers or an Achilles' heel?. Nat Rev Cancer.

[7] St-Pierre J, Drori S, Uldry M, Silvaggi JM, Rhee J, Jager S, Handschin C, Zheng K, Lin J, Yang W, Simon DK, Bachoo R, Spiegelman BM (2006). Suppression of reactive oxygen species and neurodegeneration by the PGC-1 transcriptional coactivators. Cell.

[8] Lin J, Handschin C, Spiegelman BM (2005). Metabolic control through the PGC-1 family of transcription coactivators. Cell Metabol.

[9] Scarpulla RC (2011). Metabolic control of mitochondrial biogenesis through the PGC-1 family regulatory network. Biochim Biophys Acta.

[10] Vazquez F, Lim JH, Chim H, Bhalla K, Girnun G, Pierce K, Clish CB, Granter SR, Widlund HR, Spiegelman BM, Puigserver P (2013). PGC1alpha expression defines a subset of human melanoma tumors with increased mitochondrial capacity and resistance to oxidative stress. Cancer Cell.

[11] LeBleu VS, O'Connell JT, Gonzalez Herrera KN, Wikman H, Pantel K, Haigis MC, de Carvalho FM, Damascena A, Domingos Chinen LT, Rocha RM, Asara JM, Kalluri R (2014). PGC-1alpha mediates mitochondrial biogenesis and oxidative phosphorylation in cancer cells to promote metastasis. Nat Cell Biol.

[12] Martinez-Outschoorn UE, Pavlides S, Sotgia F, Lisanti MP (2011). Mitochondrial biogenesis drives tumor cell proliferation. Am J Pathol.

[13] Mayevsky A (2009). Mitochondrial function and energy metabolism in cancer cells: past overview and future perspectives. Mitochondrion.

[14] Lowe KA, Chia VM, Taylor A, O'Malley C, Kelsh M, Mohamed M, Mowat FS, Goff B (2013). An international assessment of ovarian cancer incidence and mortality. Gynecol Oncol.

[15] Kipps E, Tan DS, Kaye SB (2013). Meeting the challenge of ascites in ovarian cancer: new avenues for therapy and research. Nat Rev Cancer.

[16] Ayantunde AA, Parsons SL (2007). Pattern and prognostic factors in patients with malignant ascites: a retrospective study. Ann Oncol.

[17] Ahmed N, Stenvers KL (2013). Getting to know ovarian cancer ascites: opportunities for targeted therapy-based translational research. Front Oncol.

[18] Thibault B, Castells M, Delord JP, Couderc B (2014). Ovarian cancer microenvironment: implications for cancer dissemination and chemoresistance acquisition. Cancer Metastasis Rev.

[19] Smolle E, Taucher V, Haybaeck J (2014). Malignant ascites in ovarian cancer and the role of targeted therapeutics. Anticancer Res.

[20] Latifi A, Luwor RB, Bilandzic M, Nazaretian S, Stenvers K, Pyman J, Zhu H, Thompson EW, Quinn MA, Findlay JK, Ahmed N (2012). Isolation and characterization of tumor cells from the ascites of ovarian cancer patients: molecular phenotype of chemoresistant ovarian tumors. PLoS One.

[21] Vathipadiekal V, Saxena D, Mok SC, Hauschka PV, Ozbun L, Birrer MJ (2012). Identification of a potential ovarian cancer stem cell gene expression profile from advanced stage papillary serous ovarian cancer. PLoS One.

[22] Wartenberg M, Ling FC, Muschen M, Klein F, Acker H, Gassmann M, Petrat K, Putz V, Hescheler J, Sauer H (2003). Regulation of the multidrug resistance transporter P-glycoprotein in multicellular tumor spheroids by hypoxia-inducible factor (HIF-1) and reactive oxygen species. FASEB J.

[23] Wu Z, Puigserver P, Andersson U, Zhang C, Adelmant G, Mootha V, Troy A, Cinti S, Lowell B, Scarpulla RC, Spiegelman BM (1999). Mechanisms controlling mitochondrial biogenesis and respiration through the thermogenic coactivator PGC-1. Cell.

[24] Westermann B (2010). Mitochondrial fusion and fission in cell life and death. Nat Rev Mol Cell Biol.

[25] Silva IA, Bai S, McLean K, Yang K, Griffith K, Thomas D, Ginestier C, Johnston C, Kueck A, Reynolds RK, Wicha MS, Buckanovich RJ (2011). Aldehyde dehydrogenase in combination with CD133 defines angiogenic ovarian cancer stem cells that portend poor patient survival. Cancer Res.

[26] Di J, Duiveman-de Boer T, Zusterzeel PL, Figdor CG, Massuger LF, Torensma R (2013). The stem cell markers Oct4A, Nanog and c-Myc are expressed in ascites cells and tumor tissue of ovarian cancer patients. Cell Oncol.

[27] Yu SC, Ping YF, Yi L, Zhou ZH, Chen JH, Yao XH, Gao L, Wang JM, Bian XW (2008). Isolation and characterization of cancer stem cells from a human glioblastoma cell line U87. Cancer Lett.

[28] Huang P, Kishida S, Cao D, Murakami-Tonami Y, Mu P, Nakaguro M, Koide N, Takeuchi I, Onishi A, Kadomatsu K (2011). The neuronal differentiation factor NeuroD1 downregulates the neuronal repellent factor Slit2 expression and promotes cell motility and tumor formation of neuroblastoma. Cancer Res.

[29] Zhang S, Balch C, Chan MW, Lai HC, Matei D, Schilder JM, Yan PS, Huang TH, Nephew KP (2008). Identification and characterization of ovarian cancer-initiating cells from primary human tumors. Cancer Res.

[30] Hashimoto N, Tsunedomi R, Yoshimura K, Watanabe Y, Hazama S, Oka M (2014). Cancer stem-like sphere cells induced from de-differentiated hepatocellular carcinoma-derived cell lines possess the resistance to anti-cancer drugs. BMC Cancer.

[31] Gao P, Zhang H, Dinavahi R, Li F, Xiang Y, Raman V, Bhujwalla ZM, Felsher DW, Cheng L, Pevsner J, Lee LA, Semenza GL, Dang CV (2007). HIF-dependent antitumorigenic effect of antioxidants in vivo. Cancer Cell.

[32] Rohwer N, Cramer T (2011). Hypoxia-mediated drug resistance: novel insights on the functional interaction of HIFs and cell death pathways. Drug Resist Updat.

[33] Qu Y, Oyan AM, Liu R, Hua Y, Zhang J, Hovland R, Popa M, Liu X, Brokstad KA, Simon R, Molven A, Lin B, Zhang WD (2013). Generation of prostate tumor-initiating cells is associated with elevation of reactive oxygen species and IL-6/STAT3 signaling. Cancer Res.

[34] Monticone M, Taherian R, Stigliani S, Carra E, Monteghirfo S, Longo L, Daga A, Dono M, Zupo S, Giaretti W, Castagnola P (2014). NAC, tiron and trolox impair survival of cell cultures containing glioblastoma tumorigenic initiating cells by inhibition of cell cycle progression. PLoS One.

[35] Zhou D, Shao L, Spitz DR (2014). Reactive oxygen species in normal and tumor stem cells. Adv Cancer Res.

[36] Tome ME, Frye JB, Coyle DL, Jacobson EL, Samulitis BK, Dvorak K, Dorr RT, Briehl MM (2012). Lymphoma cells with increased anti-oxidant defenses acquire chemoresistance. Exp Ther Med.

[37] Kumar AP, Loo SY, Shin SW, Tan TZ, Eng CB, Singh R, Putti TC, Ong CW, Salto-Tellez M, Goh BC, Park JI, Thiery JP, Pervaiz S, Clement MV (2014). Manganese superoxide dismutase is a promising target for enhancing chemosensitivity of basal-like breast carcinoma. Antioxid Redox Signal.

[38] Chen PM, Cheng YW, Wu TC, Chen CY, Lee H (2015). MnSOD overexpression confers cisplatin resistance in lung adenocarcinoma via the NF-kappaB/Snail/Bcl-2 pathway. Free Radic Biol Med.

[39] Gabrielson M, Bjorklund M, Carlson J, Shoshan M (2014). Expression of mitochondrial regulators PGC1alpha and TFAM as putative markers of subtype and chemoresistance in epithelial ovarian carcinoma. PLoS One.

[40] Oliva CR, Moellering DR, Gillespie GY, Griguer CE (2011). Acquisition of chemoresistance in gliomas is associated with increased mitochondrial coupling and decreased ROS production. PLoS One.

[41] Cai FF, Xu C, Pan X, Cai L, Lin XY, Chen S, Biskup E (2016). Prognostic value of plasma levels of HIF-1a and PGC-1a in breast cancer. Oncotarget.

[42] Ichida M, Nemoto S, Finkel T (2002). Identification of a specific molecular repressor of the peroxisome proliferator-activated receptor gamma Coactivator-1 alpha (PGC-1alpha). J Biol Chem.

