# Supplementary material for: PGC1α induced by reactive oxygen species contributes to chemoresistance of ovarian cancer cells
Source: Oncotarget. 2017 Jul 10;8(36):60299–311. doi: 10.18632/oncotarget.19140 (PMC5601140; doi:10.18632/oncotarget.19140)
Supplement: Supplementary file 1 [file oncotarget-08-60299-s001.pdf]

PGC1α induced by reactive oxygen species contributes to chemoresistance of ovarian cancer cells

SUPPLEMENTARY MATERIALS

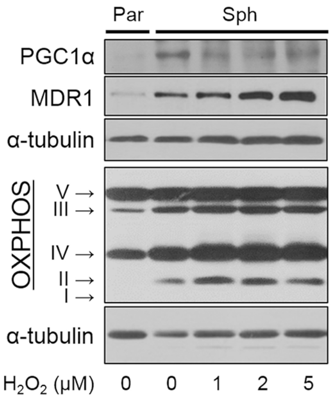

Supplementary Figure 1: Exogenous H_2O_2 addition to the spheres did not increase PGC1α expression. After serial concentrations of H_2O_2 treatment to the spheres for 48 h, expression of PGC1α, MDR1, and OXPHOS complex proteins were analyzed by Western blot.

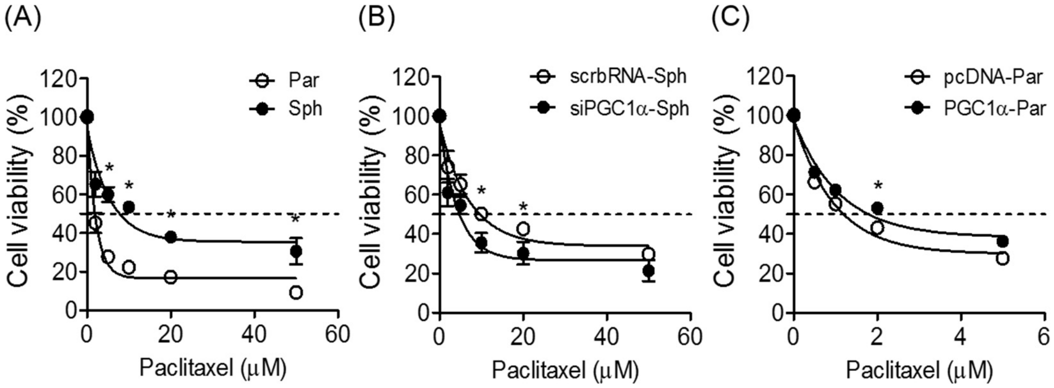

Supplementary Figure 2: Comparison of cell viability among parent, PGC1α-overexpressing parent, sphere, and PGC1α-silencing sphere cells following paclitaxel treatment for 48 h. (A) Cell viability of parent cells and spheres. **(B)** Cell viability of scrambled RNA-transfected and PGC1α-silencing spheres. **(C)** Cell viability of pcDNA-transfected and PGC1α-overexpressing parent cells ($p<0.05$).

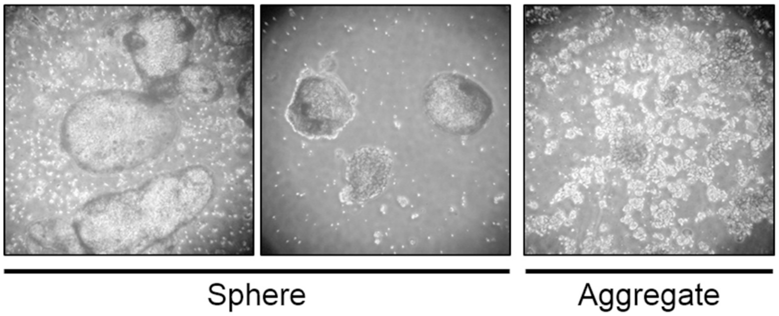

Supplementary Figure 3: Morphologies of ascites-derived cancer cells.
